# Prognostic Impact of the Lung Immune Prognostic Index in Metastatic Non-Small Cell Lung Cancer Patients Treated with Nivolumab

**DOI:** 10.3390/cancers18071170

**Published:** 2026-04-05

**Authors:** Şahin Bedir, Tanju Kapağan, Yakup Bozkaya, Abdilkerim Oyman, Mehmet Cem Fidan, Murad Guliyev, Hamza Abbasov, Nebi Serkan Demirci, Hale Gülçin Yıldırım Doğan, Emir Çelik, Nilüfer Bulut, Gökmen Umut Erdem

**Affiliations:** 1 Gaziosmanpaşa Hastanesi, Istinye University, 34010 Istanbul, Turkey; dr_yakupbozkaya@hotmail.com (Y.B.); droyman84@gmail.com (A.O.); 2 İstanbul Başakşehir Çam ve Sakura Şehir Hastanesi, 34480 Istanbul, Turkey; tanjukapagan2016@gmail.com (T.K.); ferlut@gmail.com (N.B.); gokmenumut@hotmail.com (G.U.E.); 3 Cerrahpasa Medical Faculty Hospital, Istanbul University-Cerrahpasa, 34320 Istanbul, Turkey; mcemfidan@hotmil.com (M.C.F.); drguliyev892@gmail.com (M.G.); hamzaabbasov90@gmail.com (H.A.); drserkannebi@yahoo.com (N.S.D.); 4 İstanbul Prof. Dr. Cemil Taşcioğlu Şehir Hastanesi, 34384 Istanbul, Turkey; hgulcinyildirim@hotmail.com; 5 Topkapi Liv Hospital, Faculty of Medicine, Istinye University, 34010 Istanbul, Turkey; emircelikk@gmail.com

**Keywords:** non-small cell lung cancer, immune checkpoint inhibitors, nivolumab, lung immune prognostic index, real-world data, prognosis

## Abstract

**Background**: Immune checkpoint inhibitors (ICIs) have become a cornerstone in the treatment of advanced non-small cell lung cancer (NSCLC), yet substantial heterogeneity in clinical outcomes persists. Easily accessible biomarkers that can reliably stratify prognosis in real-world practice are still lacking. The Lung Immune Prognostic Index (LIPI), integrating lactate dehydrogenase (LDH) and the derived neutrophil-to-lymphocyte ratio (dNLR), has emerged as a promising candidate, yet its clinical relevance remains incompletely defined. **Methods**: We conducted a multicenter retrospective analysis of 211 patients with metastatic NSCLC treated with second-line nivolumab between 2017 and 2025. Patients were categorized into three groups (good, intermediate, and poor) according to their LIPI score, and the relationships between LIPI and objective response, disease control, progression-free survival (PFS), and overall survival (OS) were analyzed. **Results**: LIPI stratification effectively discriminated patients into prognostically distinct groups. Objective response rates were comparable across LIPI categories and did not differ significantly. In contrast, disease control declined progressively with worsening LIPI scores, reaching statistical significance (*p* < 0.001). Overall survival was significantly worse in patients with poor LIPI, demonstrating a clear stepwise reduction from good to poor LIPI groups (*p* = 0.007). Although progression-free survival showed a consistent numerical decrease across LIPI categories, this trend did not achieve statistical significance. **Conclusions**: In patients with metastatic NSCLC receiving second-line nivolumab in a real-world setting, LIPI reliably stratified overall survival and disease control outcomes, despite limited association with early response or progression-free survival. Its simplicity and reliance on routinely available laboratory parameters support its use as a clinically meaningful prognostic tool in everyday practice.

## Simple Summary

Patients with advanced lung cancer who receive immunotherapy can experience very different outcomes, and simple tools are needed for clinicians to better predict prognosis in daily practice. This study investigates whether a blood-based score that combines routine laboratory values can help identify patients who are more likely to experience favorable clinical outcomes during treatment with nivolumab after previous therapy. By analyzing real-world data from multiple centers, we found that this score can meaningfully distinguish patients with better survival and disease control from those with poorer outcomes, even though it does not strongly predict early tumor shrinkage. Because the score relies on widely available and inexpensive tests, it may provide an easy way for clinicians to improve risk assessment and treatment planning, while also helping researchers design more tailored studies.

## 1. Introduction

Lung cancer continues to be the leading cause of cancer-related death worldwide and represents a substantial public health challenge. Data from the GLOBOCAN 2022 report estimate that nearly 2.48 million individuals were newly diagnosed with lung cancer globally in 2022 [1]. Non-small cell lung cancer (NSCLC) is the predominant histological subtype and accounts for the vast majority of cases [2].

Over the past decade, immunotherapy has significantly reshaped the therapeutic landscape of metastatic NSCLC. Immune checkpoint inhibitors (ICIs), particularly antibodies directed against programmed cell death protein-1 (PD-1) and programmed death-ligand 1 (PD-L1), are now widely used as standard treatment options, either alone or combined with chemotherapy [3]. Nevertheless, clinical outcomes remain highly variable, with some patients achieving durable benefit while others experience limited response. This variability highlights the ongoing need for practical and reliable biomarkers that can help identify patients most likely to benefit from ICI therapy [4].

Among the biomarkers investigated, lactate dehydrogenase (LDH), which reflects tumor burden and aggressive disease biology, and the derived neutrophil-to-lymphocyte ratio (dNLR), a marker of systemic inflammatory and immune status, have both been associated with prognosis in metastatic NSCLC. To improve risk stratification, these two parameters were combined to create the Lung Immune Prognostic Index (LIPI). This composite score has been proposed as a clinically useful tool to classify patients receiving immunotherapy into prognostic groups with distinct survival outcomes and treatment responses [5,6].

In this context, the present study aimed to assess the prognostic value of LIPI in patients with metastatic NSCLC treated with second-line nivolumab. We specifically examined the relationship between LIPI classification and treatment response, as well as progression and survival outcomes, to determine whether LIPI can serve as a practical biomarker for prognostic stratification in routine clinical practice.

## 2. Materials and Methods

### 2.1. Study Design and Patient Selection

This retrospective cohort study was conducted using data from patients diagnosed with metastatic non-small cell lung cancer (mNSCLC) who received nivolumab monotherapy as second-line treatment between January 2017 and November 2025. Patients were treated at the Medical Oncology departments of Istinye University Gaziosmanpaşa Hospital, Başakşehir Çam and Sakura City Hospital, and Istanbul University–Cerrahpaşa Faculty of Medicine Hospital.

### 2.2. Inclusion Criteria

Eligible patients fulfilled the following criteria:

1. Histological or cytological confirmation of non-small cell lung cancer (NSCLC);
2. Metastatic disease at the time of nivolumab initiation (stage IV according to the 8th edition of the TNM classification);
3. Treatment with nivolumab administered as monotherapy in the second-line setting;
4. Availability of baseline laboratory data prior to treatment initiation, including complete blood count and biochemical parameters, particularly neutrophil, lymphocyte, total white blood cell counts, and serum lactate dehydrogenase (LDH) levels;
5. Age ≥ 18 years at the time of treatment.

### 2.3. Exclusion Criteria

Patients presenting with one or more of the following conditions were excluded from the study:

1. A diagnosis of small cell lung cancer or mixed histology (NSCLC with a small cell component);
2. Prior treatment with immune checkpoint inhibitors;
3. Unavailability of pretreatment LDH or dNLR data;
4. Presence of a concomitant malignancy;
5. A follow-up duration of less than three months.

### 2.4. Follow-Up Requirement

A minimum follow-up of three months was applied to ensure standardized radiological response assessment, aligning with the institutional protocol of performing the first tumor evaluation at 12 weeks. Patients with shorter follow-up were excluded due to incomplete clinical records or loss to follow-up, ensuring a robust cohort for response and survival analysis.

### 2.5. Clinical and Pathological Assessment

Clinical and pathological data for all patients were retrospectively obtained from the electronic medical record system. Clinical variables included age, sex, smoking history, histological subtype, sites of metastasis, and treatment regimens. Histopathological diagnosis was established based on biopsy specimens obtained from either the primary tumor or metastatic lesions. Tumors were classified as adenocarcinoma, squamous cell carcinoma, or other rare subtypes according to the 2021 World Health Organization (WHO) Classification of Lung Tumors [7]. Disease staging was performed in accordance with the eighth edition of the TNM classification system proposed by the International Association for the Study of Lung Cancer (IASLC) [8]. The diagnosis of metastatic disease was confirmed using positron emission tomography/computed tomography (PET/CT), thoracic and abdominal CT scans, and brain magnetic resonance imaging (MRI). Molecular analyses performed prior to treatment were used to determine the presence of EGFR mutations, ALK rearrangements, ROS1 fusions, and KRAS mutations in tumor tissue.

PD-L1 expression was assessed on tumor tissue using immunohistochemistry (IHC) according to institutional practice at each participating center. PD-L1 tumor proportion score (TPS) was defined as the percentage of viable tumor cells showing membranous staining. Patients were categorized as TPS < 1%, TPS 1–49%, or TPS ≥ 50% for analysis.

Pretreatment laboratory parameters, including absolute neutrophil, lymphocyte, and total white blood cell counts, serum lactate dehydrogenase (LDH), C-reactive protein (CRP), and albumin levels, were recorded. For the purposes of analysis, CRP was categorized as ≤10 mg/L versus >10 mg/L, and albumin as ≥3.5 g/dL versus <3.5 g/dL.

### 2.6. Treatment and Response Assessment

Nivolumab was administered intravenously at either 3 mg/kg or as a flat dose of 240 mg every two weeks in accordance with institutional practice [9]. Treatment was maintained until radiological disease progression or unacceptable adverse events occurred. Radiologic evaluations were performed at 12-week intervals using computed tomography (CT) or positron emission tomography/computed tomography (PET/CT). Tumor response was assessed according to the Response Evaluation Criteria in Solid Tumors (RECIST) version 1.1 [10], the objective response rate (ORR) and the disease control rate (DCR) were calculated based on these criteria.

RECIST measurements were based on the anatomical CT component of imaging studies. When PET/CT was performed as part of routine clinical practice, metabolic response criteria were not used for response classification. Patients were considered evaluable for response if baseline imaging and at least one post-baseline radiological assessment were available after the initiation of nivolumab therapy.

### 2.7. Calculation of the Lung Immune Prognostic Index (LIPI)

The Lung Immune Prognostic Index (LIPI) was calculated using two baseline laboratory parameters obtained prior to the initiation of nivolumab therapy:

1. Derived neutrophil-to-lymphocyte ratio (dNLR), calculated as the absolute neutrophil count divided by the difference between the total white blood cell count and the neutrophil count;
2. Serum lactate dehydrogenase (LDH) level.

Patients were stratified into three prognostic groups according to the classification proposed by Mezquita et al. [6]:

• **Good LIPI:** dNLR ≤ 3 and normal LDH levels;
• **Intermediate LIPI:** impairment of one parameter (dNLR > 3 or elevated LDH);
• **Poor LIPI:** impairment of both parameters (dNLR > 3 and elevated LDH).

Serum LDH levels were interpreted according to the upper limit of normal (ULN) defined by the local laboratory at each participating center, and LDH elevation was defined as a value above this threshold. Baseline laboratory parameters used for LIPI calculation were obtained prior to nivolumab initiation, and when multiple measurements were available, the value closest to the start of treatment was selected.

In addition to LIPI, other inflammation-based prognostic scores, including the Glasgow Prognostic Score (GPS), Prognostic Nutritional Index (PNI), and Neutrophil–Platelet Score (NPS), were calculated using baseline laboratory parameters. GPS was defined based on serum C-reactive protein and albumin levels, PNI was calculated as 10 × serum albumin (g/dL) + 0.005 × lymphocyte count (/mm^3^), and NPS was derived from predefined neutrophil and platelet thresholds.

For the purposes of statistical analysis, these indices were categorized as follows: GPS was grouped as 0 versus 1–2, NPS as 0 versus 1–2, and PNI was dichotomized as high versus low according to the predefined cutoff value used in the analysis. The cutoff value for PNI was set at 46.5, based on receiver operating characteristic (ROC) curve analysis.

### 2.8. Outcome Measures

The disease control rate (DCR) was defined as the percentage of patients who achieved complete response, partial response, or stable disease lasting at least six months after initiation of nivolumab therapy. The objective response rate (ORR) represented the percentage of patients with either complete or partial response. Overall survival (OS) was measured from the start of nivolumab treatment until death from any cause or the last follow-up visit. Progression-free survival (PFS) was defined as the time from the initiation of nivolumab therapy to the first occurrence of radiologically confirmed disease progression or death from any cause, whichever occurred first. Patients who were alive and without documented progression at the last follow-up were censored at the date of last contact.

### 2.9. Statistical Analysis

Patient demographics and clinical characteristics were summarized using descriptive statistics. Continuous variables were reported as mean ± standard deviation or median (range), depending on their distribution, while categorical variables were expressed as counts and percentages. Survival outcomes were analyzed using the Kaplan–Meier method, and differences between groups were evaluated with the log-rank test. Univariate and multivariate Cox proportional hazards regression analyses were conducted to identify independent prognostic factors associated with overall survival (OS) and progression-free survival (PFS). The proportional hazards assumption for the Cox regression models was evaluated using graphical inspection of log-minus-log survival plots. No missing data were present for the variables included in the analyses. Variables included in the analyses were LIPI, GPS, PNI, NPS, age, sex, histology, sites of metastasis (including visceral involvement), CRP, and albumin. Logistic regression was used to evaluate associations with the objective response rate (ORR) and disease control rate (DCR).

The association of these indices with survival outcomes was evaluated using univariate and multivariate Cox proportional hazards regression analyses.

To compare the discriminatory performance of these prognostic scores, receiver operating characteristic (ROC) curve analysis was performed at 12 months, and the area under the curve (AUC) was calculated for each score.

A two-sided *p* value of less than 0.05 was considered statistically significant. All analyses were performed using IBM SPSS Statistics software (version 26.0; IBM Corp., Armonk, NY, USA).

This study was conducted and reported in accordance with the Strengthening the Reporting of Observational Studies in Epidemiology (STROBE) guidelines and followed the general principles of the Reporting Recommendations for Tumor Marker Prognostic Studies (REMARK).

### 2.10. Ethics Approval

The study protocol was reviewed and approved by the Clinical Research Ethics Committee of Yeni Yüzyıl University Faculty of Medicine (Approval No: [2026/01-1814]; Date: [13 January 2026]). Given the retrospective nature of the study and the use of anonymized patient records, informed consent was waived. All procedures were conducted in accordance with the ethical standards set forth in the 2013 revision of the Declaration of Helsinki [11].

## 3. Results

### 3.1. Patient Characteristics

**Figure 1:**
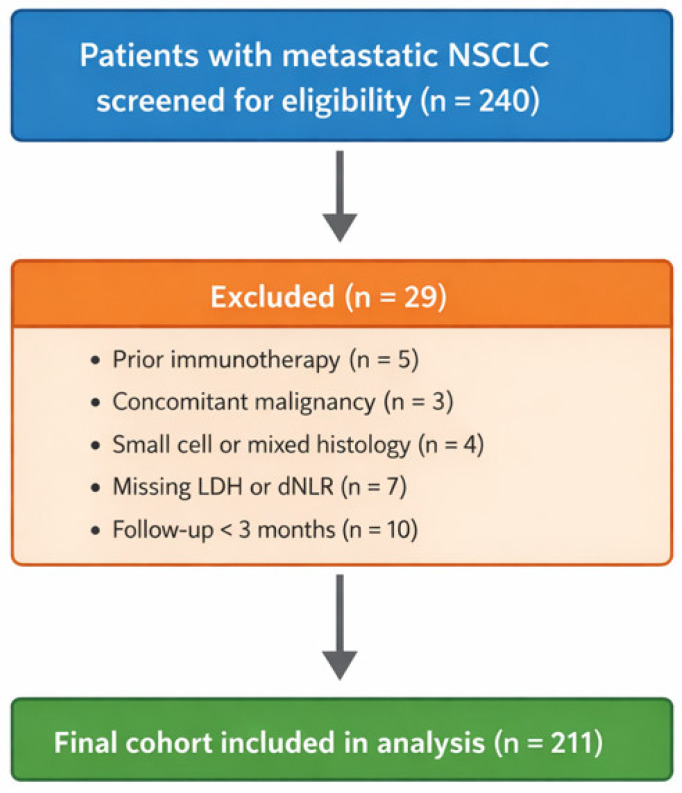
Patient flow diagram. CONSORT-style diagram showing the screening, exclusion, and inclusion of patients for the final cohort. Exclusion reasons included prior immunotherapy, concomitant malignancy, small cell or mixed histology, missing LDH or dNLR data, and follow-up < 3 months.

A total of 240 patients with metastatic NSCLC who received nivolumab were initially screened. After applying the predefined inclusion and exclusion criteria, 211 patients were included in the final analysis. The patient selection process and reasons for exclusion are summarized in Figure 1.

The median age of the study population was 63 years (range, 23–84), with 94 patients (44.5%) aged 65 years or older. Of the patients, 176 (83.4%) were male, and 185 (87.7%) were smokers. ECOG performance status was 0–1 in 178 patients (84.4%), while 33 patients (15.6%) had an ECOG performance status of 2. Histopathological evaluation revealed non-squamous carcinoma in 138 cases (65.4%) and squamous cell carcinoma in 73 cases (34.6%). De novo metastatic disease was observed in 122 patients (58.2%), while recurrent disease was identified in 89 patients (41.8%). Regarding metastatic patterns, visceral metastases were detected in 87 patients (41.2%), including liver metastases in 29 patients (13.7%) and brain metastases in 39 patients (18.5%), whereas non-visceral metastases were present in 124 patients (58.8%), including bone metastases in 70 patients (33.2%). PD-L1 expression status was classified as high (tumor proportion score [TPS] ≥ 50) in 47 patients (22.3%), intermediate (TPS 1–49) in 71 patients (33.7%), and negative (TPS < 1) in 93 patients (44.1%). Serum albumin levels were ≥3.5 g/dL in 186 patients (88.2%), and CRP levels were >10 mg/L in 139 patients (65.9%).

According to LIPI stratification, 67 patients (31.75%) were classified as having good LIPI, 109 patients (51.65%) as having intermediate LIPI, and 35 patients (16.6%) as having poor LIPI. Patients in the poor LIPI group exhibited significantly higher rates of visceral metastasis (*p* = 0.046), liver metastasis (*p* = 0.021), elevated CRP (*p* = 0.042), and ECOG PS 2 (*p* = 0.040), while other clinicopathological variables showed no significant differences (Table 1).

### 3.2. Response Rates

All patients received nivolumab as second-line therapy for advanced non-small cell lung cancer. Among the 211 patients evaluable for treatment response, the objective response rate (ORR) was 38.8% in the good LIPI group, 33.9% in the intermediate group, and 20% in the poor group, with no statistically significant differences observed between the groups (OR = 1.488; 95% CI: 0.964–2.297; *p* = 0.073). In contrast, the disease control rate (DCR) decreased progressively with worsening LIPI scores, reaching 76.1% in the good group, 60.6% in the intermediate group, and 37.1% in the poor group. This stepwise decline in DCR across LIPI categories was statistically significant (OR = 2.295; 95% CI: 1.479–3.561; *p* < 0.001). A summary of treatment response rates is provided in Table 2.

### 3.3. Progression-Free Survival (PFS)

**Figure 2:**
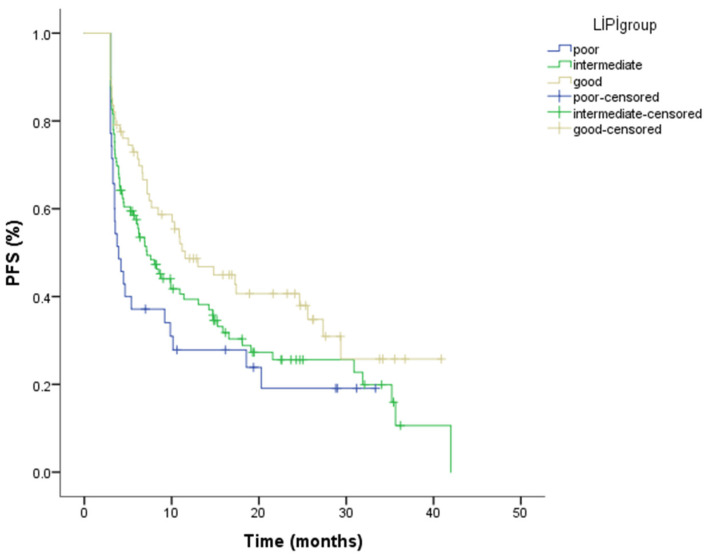
Kaplan–Meier curves for progression-free survival according to Lung Immune Prognostic Index (LIPI) groups, demonstrating a statistically significant difference in PFS among the groups (log-rank *p* = 0.037). The number of patient at risk at selected time points is shown below the Kaplan–Meier curves.

The median follow-up for the cohort was 12.05 months (range: 3.09–57.59). During follow-up, 146 PFS events occurred, including both radiologically documented disease progression and deaths without prior documented progression. For the entire population, the median progression-free survival (PFS) was 8.49 months (95% CI: 5.83–10.26). When stratified by LIPI score, the median PFS was 11.53 months (95% CI: 6.26–16.79) in the good LIPI group, 7.16 months (95% CI: 4.96–9.35) in the intermediate group, and 3.94 months (95% CI: 2.76–5.12) in the poor group. Kaplan–Meier analysis indicated statistically significant difference in PFS between LIPI categories (*p* = 0.037) (Figure 2; Table 3). The number of patients at risk for progression-free survival at selected time points for each LIPI group is presented in Table 4.

In the univariate Cox regression analysis, female sex, liver metastasis, bone metastasis, low PD-L1 expression, hypoalbuminemia, elevated CRP levels, high GPS, low PNI, high NPS and poor LIPI group were associated with shorter progression-free survival. However, in the multivariate analysis, only female sex (HR = 2.41, 95% CI: 1.11–5.21, *p* = 0.025), lower PD-L1 expression (≥50% vs. <1%: HR = 3.17, 95% CI: 1.32–7.57, *p* = 0.009; ≥50% vs. 1–49%: HR = 2.48, 95% CI: 1.06–5.80, *p* = 0.036), and elevated CRP levels (HR = 2.14, 95% CI: 1.10–4.17, *p* = 0.023) remained independently associated with shorter progression-free survival, whereas the association between LIPI groups and PFS did not remain statistically significant after adjustment for confounding variables (Table 4).

### 3.4. Overall Survival (OS)

**Figure 3:**
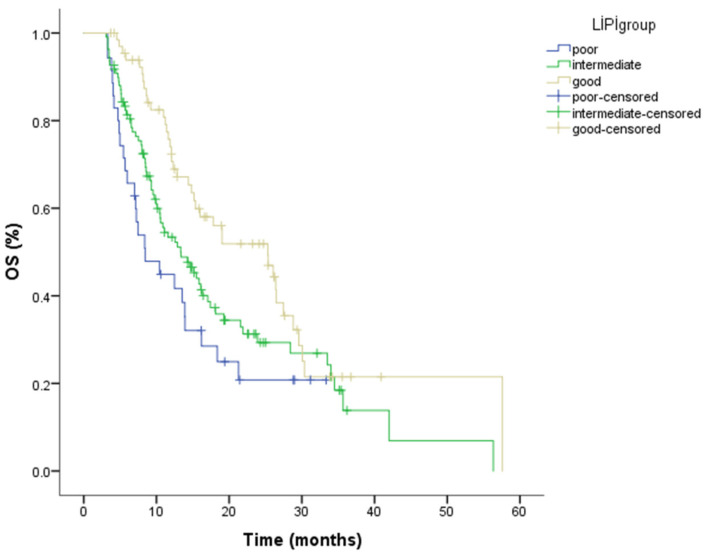
Kaplan–Meier curves for overall survival stratified by Lung Immune Prognostic Index (LIPI) groups, demonstrating a statistically significant difference in OS among the groups (log-rank *p* = 0.007). The number of patients at risk at selected time points is shown below the Kaplan–Meier curves.

During follow-up, 137 patients (64.9%) died. The median overall survival (OS) for the entire cohort was 14.58 months (95% CI: 12.34–16.82). When analyzed by LIPI category, median OS was 25.33 months (95% CI: 15.96–34.69) in the good LIPI group, 13.33 months (95% CI: 9.35–17.31) in the intermediate group, and 8.44 months (95% CI: 3.99–12.89) in the poor group. Kaplan–Meier analysis revealed a statistically significant difference in OS across LIPI groups (*p* = 0.007) (Figure 3; Table 5). The numbers of patients at risk for overall survival at selected time points for each LIPI group are presented in Table 6.

In the univariate Cox regression analysis, albumin level, CRP level, PD-L1 expression, GPS, PNI, NPS and LIPI group were significantly associated with overall survival. In the multivariate analysis, lower PD-L1 expression (<1% vs. ≥50%: HR = 2.88, 95% CI: 1.25–6.65, *p* = 0.013), low albumin levels (HR = 3.16, 95% CI: 1.18–7.65, *p* = 0.016), and poor LIPI status (poor vs. good: HR = 2.53, 95% CI: 1.32–6.38, *p* = 0.034) remained independently associated with shorter overall survival, whereas CRP level and bone metastasis did not retain statistical significance after adjustment (Table 7).

Receiver operating characteristic (ROC) analysis was performed to evaluate the discriminatory performance of the inflammation-based prognostic scores. The AUC for LIPI was 0.605, indicating moderate discriminatory ability. In comparison, the AUC values were 0.576 for GPS, 0.560 for PNI, and 0.549 for NPS, suggesting overall modest performance for these indices. Among all evaluated scores, LIPI demonstrated the highest discriminatory accuracy (Table 8).

## 4. Discussion

Despite the clinical benefits conferred by immune checkpoint inhibitors (ICIs) in the treatment of advanced non-small cell lung cancer (NSCLC), the accurate identification of patients who are most likely to benefit remains a critical clinical challenge. Although PD-L1 expression assessed by immunohistochemistry in tumor tissue initially emerged as a promising biomarker, its predictive utility in clinical practice is limited due to intratumoral heterogeneity, dynamic changes in expression, and methodological variability. In this context, there is growing interest in readily accessible biomarkers that reflect systemic tumor–host interactions. Considering the pivotal role of real-world data in biomarker validation, this study evaluated the association of the Lung Immune Prognostic Index (LIPI) with treatment response and survival outcomes in a cohort of patients with advanced NSCLC receiving second-line nivolumab, in order to assess its prognostic significance.

In our cohort, patients with poor LIPI scores exhibited a higher prevalence of visceral metastases, suggesting a link between adverse LIPI status and more aggressive disease biology. This finding is biologically plausible, as elevated lactate dehydrogenase reflects increased tumor burden and enhanced tumor metabolic activity, whereas a high derived neutrophil-to-lymphocyte ratio indicates a systemic inflammatory environment that may facilitate tumor progression and dissemination. Consistent with this interpretation, previous studies have shown that poor LIPI scores are associated with a greater number of metastatic sites at baseline and worse survival outcomes, including both overall survival (OS) and progression-free survival (PFS), supporting the notion that LIPI primarily reflects underlying tumor aggressiveness rather than solely predicting response to immunotherapy [12].

With respect to treatment response, objective response rates differed numerically across LIPI categories, with rates of 38.8%, 33.9%, and 20% observed in the good, intermediate, and poor LIPI groups, respectively; however, these differences did not reach statistical significance. Similar findings have been reported by Ruiz Bañobre and Zhi et al., who also did not observe a significant association between LIPI classification and objective response rates [13,14]. In contrast, a large cohort analysis by Sorich et al. reported a significant association between LIPI and ORR [15]. Differences across studies may partly reflect variations in study populations and treatment strategies, as previous analyses often included heterogeneous cohorts receiving immunotherapy in different treatment lines, various checkpoint inhibitors, or combination regimens, whereas our study focused exclusively on patients treated with second-line nivolumab.

In contrast to the ORR, the disease control rate (DCR) differed significantly according to LIPI classification, showing a clear stepwise decrease from the good to the poor LIPI groups (76.1%, 60.6%, and 37.1%, respectively). This pattern suggests that worsening LIPI scores are associated with reduced clinical benefit from immunotherapy. Similar observations have been reported in prior studies, including that by Ruiz Bañobre et al., where patients with poor LIPI scores demonstrated significantly lower disease control rates compared to other LIPI subgroups [13,14].

Further analyses of progression-free survival (PFS) revealed several clinical and biological factors associated with treatment outcomes. In our cohort, male sex was significantly associated with longer PFS. A similar observation was reported by Calleja-Chucla et al., who also found that male patients experienced longer PFS in the context of immunotherapy-treated NSCLC [16]. In contrast, a real-world study by Knetki-Wróblewska et al. did not identify a significant relationship between sex and PFS [17]. These discrepancies may largely reflect differences in patient populations and study designs. Notably, the study by Knetki-Wróblewska et al. included a heterogeneous cohort of patients receiving second-line nivolumab or atezolizumab, and reported that tumor burden, inflammatory parameters, and metastatic patterns had a stronger influence on PFS than demographic characteristics.

PD-L1 expression also demonstrated prognostic relevance in our analysis. Patients with higher PD-L1 expression exhibited significantly longer PFS. Similar findings were reported by Jingyaa Liu et al., who observed significantly prolonged PFS in patients with PD-L1 expression ≥ 1% [18]. However, other studies have reported inconsistent results. For example, Brahmer et al. and Lang et al. did not observe a significant association between PD-L1 expression and PFS [9,19]. Such discrepancies may arise from heterogeneity in patient populations, differences in treatment lines, variations in PD-L1 assessment thresholds, and the use of different immune checkpoint inhibitors across studies.

Systemic inflammatory markers significantly influenced treatment outcomes in our cohort, with elevated baseline CRP levels associated with both shorter PFS and poorer LIPI scores (*p* = 0.042). These findings are consistent with previous reports demonstrating that systemic inflammatory status, particularly baseline CRP levels, is a critical prognostic determinant of survival outcomes in NSCLC patients receiving immune checkpoint inhibitors [20,21,22]. Collectively, our data reinforce the notion that systemic inflammation plays a pivotal role in shaping clinical responses to immunotherapy.

Although a numerical trend toward shorter PFS was observed with worsening LIPI categories in our cohort, this difference did not reach statistical significance. Similar observations have been reported by Ruiz-Bañobre et al. and Huang et al., who described longer PFS in patients with favorable LIPI scores, although the differences did not achieve statistical significance [13,23]. In contrast, Zhi et al. and Sorich et al. demonstrated a significant association between poor LIPI scores and shorter PFS in patients with advanced NSCLC [14,15]. These inconsistencies may partly reflect differences in treatment regimens across studies. Many previous analyses included patients receiving both PD-1 and PD-L1 inhibitors and, in some cases, combination regimens with chemotherapy. In addition, variations in baseline disease burden and performance status may also contribute to the heterogeneity observed in the prognostic impact of LIPI.

With respect to overall survival (OS), higher PD-L1 expression was significantly associated with improved survival outcomes in our cohort. Similarly, Borghaei et al. reported that PD-L1 expression was associated with longer OS in patients with advanced NSCLC receiving second-line nivolumab [24]. However, other studies have reported conflicting results. For example, Brahmer et al. and Lang et al. did not observe a significant association between PD-L1 expression and OS [9,19]. These discrepancies likely reflect differences in patient populations, treatment lines, and clinical characteristics across studies. In particular, several reports have suggested that clinical factors such as performance status and treatment line may exert a stronger influence on survival outcomes than PD-L1 expression alone.

Serum albumin level also emerged as a significant prognostic factor for OS in our study, with higher baseline albumin levels associated with improved survival. This finding is consistent with prior evidence indicating that baseline serum albumin is associated with OS and may function as an independent prognostic marker in NSCLC patients, including those treated with immune checkpoint inhibitors [25,26,27]. Taken together, these data suggest that serum albumin, reflecting nutritional status, systemic inflammation, and overall physiological reserve, may serve as a readily available prognostic biomarker in patients undergoing immunotherapy.

Consistent with these observations, the LIPI score also demonstrated a clear prognostic impact on overall survival in our cohort. Patients with poor LIPI scores experienced significantly shorter OS compared to those with favorable scores. This finding is consistent with previous studies. Ruiz-Bañobre et al. reported that poor LIPI scores were significantly associated with shorter OS in patients with advanced NSCLC treated with nivolumab [13]. Similarly, Zhi et al. demonstrated significantly longer OS in patients with favorable LIPI scores and identified LIPI as an independent prognostic factor in multivariable analysis [14]. In addition, pooled analyses conducted by Sorich et al. across multiple clinical trials confirmed that LIPI classification was significantly associated with OS across different treatment groups [15]. Taken together, these findings support the clinical utility of LIPI as a practical and reproducible biomarker reflecting systemic inflammation and tumor biology, with potential value for prognostic stratification in NSCLC patients receiving immune checkpoint inhibitors.

In this comparative analysis, LIPI consistently demonstrated superior discriminatory performance compared to other commonly used inflammation-based prognostic scores, including GPS, PNI, and NPS. While all indices showed some degree of prognostic relevance, their overall ability to distinguish between risk groups was modest. Notably, NPS exhibited the lowest performance, whereas PNI and GPS provided intermediate discriminatory value. These findings suggest that LIPI, by integrating both inflammatory and tumor burden–related parameters, may offer a more robust tool for risk stratification in patients with advanced NSCLC receiving immune checkpoint inhibitors.

### Strengths and Limitations

This study’s main strength is its multicenter real-world design, including a relatively large cohort of patients with metastatic NSCLC uniformly treated with second-line nivolumab, which enhances the generalizability of the findings. The use of routinely available laboratory parameters for LIPI calculation supports the clinical applicability of the results.

However, several limitations should be considered. Because this study was based on retrospectively collected data, the possibility of selection bias and residual confounding cannot be completely excluded. In addition, the duration of follow-up was relatively limited, which may have reduced the statistical power to detect potential differences in survival outcomes. In addition, patients with follow-up shorter than three months were excluded to ensure adequate radiological response assessment, as the first routine imaging is typically performed around 12 weeks after treatment initiation. While this criterion improves the reliability of response evaluation, it may have introduced a minor selection bias that should be considered when interpreting survival outcomes.

## 5. Conclusions

In summary, the findings of this multicenter real-world analysis indicate that the Lung Immune Prognostic Index (LIPI) has meaningful prognostic relevance in patients with metastatic non-small cell lung cancer receiving second-line nivolumab. While LIPI classification was not significantly related to progression-free survival or objective response rate, patients with unfavorable LIPI scores experienced poorer disease control and shorter overall survival. Because LIPI is derived from routinely available laboratory parameters and can be easily implemented in clinical practice, it may be a practical approach for prognostic risk stratification in patients undergoing immunotherapy.

## Figures and Tables

**Table 1:** Description of the study population according to the Lung Immune Prognostic Index (LIPI).

Characteristic	All (N = 211), (%)	LIPI Good (N = 67), (%31.75)	LIPI Intermediate (N = 109), (%51.65)	LIPI Poor (N = 35), (%16.6)	*p*-Value
Age, median (range)	63 (23–84)	64 (34–83)	64 (23–84)	62 (31–78)	0.072
Age group					0.230
<65	117 (55.5)	35 (52.2)	58 (53.2)	24 (68.6)	
≥65	94 (44.5)	32 (47.8)	51 (46.8)	11 (31.4)	
Sex					0.112
Female	35 (16.6)	6 (9)	21 (19.3)	8 (22.9)	
Male	176 (83.4)	61 (91)	88 (80.7)	27 (77.1)	
ECOG PS					0.040
0–1	178 (84.4)	65 (97)	86 (78.9)	27 (77.1)	
2	33 (15.6)	2 (3)	23 (21.1)	8 (22.9)	
Smoking status					0.883
Yes	185 (87.7)	58 (86.6)	95 (87.2)	32 (91.4)	
No	26 (12.3)	9 (13.4)	14 (12.8)	3 (8.6)	
Histology					0.429
Non squamous	138 (65.4)	46 (68.7)	67 (61.5)	25 (71.4)	
Squamous	73 (34.6)	21 (31.3)	42 (38.5)	10 (28.6)	
Disease status					0.176
De novo metastatic	122 (58.2)	40 (59.7)	57 (52.3)	25 (71.4)	
Recurrent metastatic	89 (41.8)	27 (40.3)	52 (47.7)	10 (28.6)	
Site of metastasis					0.046
Visceral	87 (41.2)	26 (38.8)	40 (36.7)	21 (60)	
Nonvisceral	124 (58.8)	41 (61.2)	69 (63.3)	14 (40)	
Liver metastasis					0.021
No	182 (86.3)	60 (89.6)	97 (89)	25 (71.4)	
Yes	29 (13.7)	7 (10.4)	12 (11)	10 (28.6)	
Brain metastasis					0.586
No	172 (81.5)	57 (85.1)	88 (80.7)	27 (77.1)	
Yes	39 (18.5)	10 (14.9)	21 (19.3)	8 (22.9)	
Bone metastasis					0.704
No	141 (66.8)	42 (62.7)	75 (68.8)	24 (68.6)	
Yes	70 (33.2)	25 (37.3)	34 (31.2)	11 (31.4)	
PD-L1 TPS					0.927
<1%	93 (44.1)	29 (43.3)	51 (46.8)	13 (37.15)	
1–49%	71 (33.7)	25 (37.3)	33 (30.3)	13 (37.15)	
≥50%	47 (22.3)	13 (19.4)	25 (22.9)	9 (25.7)	
Albumin					0.701
≥3.5 g/dL	186 (88.2)	61 (91)	94 (86.2)	31 (88.6)	
<3.5 g/dL	25 (11.8)	6 (9)	15 (13.8)	4 (11.4)	
Crp					0.042
>10 mg/L	139 (65.9)	35 (52.2)	76 (69.7)	28 (80)	
≤10 mg/L	72 (34.1)	32 (47.8)	33 (30.3)	7 (20)	

Footnote: Data are presented as median (range) or number (%). Group comparisons were performed using the Kruskal–Wallis test for continuous variables and the χ^2^ or Fisher’s exact test for categorical variables. A *p*-value < 0.05 was considered statistically significant. LIPI was based on dNLR and LDH levels. PD-L1 TPS was assessed via immunohistochemistry.

**Table 2:** Treatment response rates in LIPI-good, LIPI-intermediate and LIPI-poor groups.

Parameter	LIPI-Good (%)	LIPI-Intermediate (%)	LIPI-Poor (%)	Odds Ratio (95% CI)	*p*-Value
Objective Response Rate (ORR)	26/67 (38.8)	37/109 (33.9)	7/35 (20)	1.488 (0.964–2.297)	0.073
Disease Control Rate (DCR)	51/67 (76.1)	66/109 (60.6)	13/35 (37.1)	2.295 (1.479–3.561)	<0.001

Footnote: The ORR was defined as the proportion of patients achieving complete or partial response, and the DCR as the proportion achieving complete response, partial response, or stable disease. Odds ratios (ORs) with 95% confidence intervals (CIs) were estimated using logistic regression with the LIPI group modeled as an ordinal variable (good → intermediate → poor). A *p*-value < 0.05 was considered statistically significant.

**Table 3:** Number of patients at risk for progression-free survival at selected time points according to LIPI group.

Time (Months)	Good	Intermediate	Poor
0	67	109	35
6	49	64	14
12	31	34	8

Footnote: The number of patients at risk represents the number of patients remaining under observation without documented progression or death at the beginning of each time point.

**Table 4:** Prognostic variables for progression-free survival in univariate analysis and multivariate analysis.

	Univariate Analysis		Multivariate Analysis	
Variables	HR (95% CI for HR)	*p* Value	HR (95% CI for HR)	*p* Value
Age in years (≥65 vs. <65)	1.13 (0.81–1.57)	0.453		
Sex (male vs. female)	1.65 (1.09–2.52)	0.018	2.41 (1.11–5.21)	0.025
ECOG PS (0–1 vs. 2)	1.04 (0.59–1.81)	0.886		
Smoking status (yes vs. never)	1.07 (0.61–1.89)	0.793		
Histology				
(Non-squamous vs. squamous)	1.14 (0.80–1.64)	0.446		
Disease status				
(Recurrent vs. de novo metastatic)	1.28 (0.90–1.81)	0.162		
Site of metastasis				
(Nonvisceral vs. visceral)	1.10 (0.79–1.53)	0.562		
Liver metastasis (no vs. yes)	1.63 (1.04–2.55)	0.033	1.17 (0.56–2.43)	0.671
Brain metastasis (no vs. yes)	1.12 (0.74–1.70)	0.579		
Bone metastasis (no vs. yes)	1.41 (1.00–1.98)	0.045	1.09 (0.62–1.92)	0.747
PD-L1 TPS		0.038		0.033
(≥50% vs. <1%)	2.42 (1.20–4.85)	0.013	3.17 (1.32–7.57)	0.009
(≥50% vs. 1–49%)	2.32 (1.12–4.78)	0.023	2.48 (1.06–5.80)	0.036
Albumin (≥3.5 g/dL vs. <3.5 g/dL)	1.89 (1.11–3.21)	0.017	2.63 (0.54–4.03)	0.230
Crp (≤10 mg/L vs. >10 mg/L)	1.79 (1.16–2.77)	0.008	2.14 (1.10–4.17)	0.023
LIPI group		0.043		0.402
(Good vs. intermediate)	1.40 (0.95–2.04)	0.082	0.98 (0.52–1.86)	0.974
(Good vs. poor)	1.83 (1.12–2.99)	0.015	1.74 (0.65–4.67)	0.267
GPS (0 vs. 1–2)	0.60 (0.38–0.93)	0.025	0.54 (0.27–1.08)	0.086
PNI (high vs. low)	1.55 (1.06–2.29)	0.024	1.31 (0.72–2.39)	0.369
NPS (0 vs. 1–2)	0.62 (0.44–0.87)	0.006	0.72 (0.36–1.45)	0.366

Footnote: HR, hazard ratio; CI, confidence interval; ECOG PS, Eastern Cooperative Oncology Group performance status; PD-L1 TPS, programmed death-ligand 1 tumor proportion score; CRP, C-reactive protein; LIPI, Lung Immune Prognostic Index; GPS, Glasgow Prognostic Score; PNI, prognostic nutritional index; NPS, neutrophil–platelet score. PFS, progression-free survival.

**Table 5:** Median PFS and OS in LIPI-good, LIPI-intermediate and LIPI-poor groups.

Parameter	LIPI-Good Months (95% CI)	LIPI-Intermediate Months (95% CI)	LIPI-Poor Months (95% CI)	*p*-Value
Progression Free Survival (PFS)	11.53 (6.26–16.79)	7.16 (4.96–9.35)	3.94 (2.76–5.12)	0.037
Overall Survival (OS)	25.33 (15.96–34.69)	13.33 (9.35–17.31)	8.44 (3.99–12.89)	0.007

Footnote: Survival outcomes are presented as median months with 95% confidence intervals (CIs). Progression-free survival (PFS) was defined as the interval from the start of nivolumab therapy to documented disease progression or death from any cause, while overall survival (OS) was calculated from treatment initiation to death from any cause. Survival differences between LIPI groups were assessed using the log-rank test. A two-sided *p*-value less than 0.05 was considered statistically significant.

**Table 6:** Number of patients at risk for overall survival at selected time points according to LIPI group.

Time (Months)	Good	Intermediate	Poor
0	67	109	35
6	63	94	26
12	48	51	14

Footnote: Numbers represent the number of patients at risk for overall survival (OS) at the indicated time points in each Lung Immune Prognostic Index (LIPI) group, based on Kaplan–Meier survival analysis.

**Table 7:** Prognostic variables for overall survival in univariate and multivariate analyses.

	Univariate Analysis		Multivariate Analysis	
Variables	HR (95% CI for HR)	*p* Value	HR (95% CI for HR)	*p* Value
Age in years (≥65 vs. <65)	1.21 (0.86–1.70)	0.252		
Sex (male vs. female)	1.19 (0.78–1.84)	0.410		
ECOG PS (0–1 vs. 2)	1.21 (0.67–2.19)	0.523		
Smoking status (yes vs. never)	1.22 (0.68–2.21)	0.493		
Histology				
(Non-squamous vs. squamous)	1.03 (0.71–1.49)	0.864		
Disease status				
(Recurrent vs. de novo metastatic)	1.14 (0.79–1.63)	0.469		
Site of metastasis				
(Nonvisceral vs. visceral)	1.09 (0.77–1.54)	0.612		
Liver metastasis (no vs. yes)	1.38 (0.87–2.20)	0.163		
Brain metastasis (no vs. yes)	1.15 (0.75–1.75)	0.516		
Bone metastasis (no vs. yes)	1.40 (0.98–1.99)	0.061	1.33 (0.74–2.36)	0.330
PD-L1 TPS		0.214		0.036
(≥50% vs. <1%)	1.86 (0.92–3.75)	0.082	2.88 (1.25–6.65)	0.013
(≥50% vs. 1–49%)	1.52 (0.72–3.20)	0.269	1.78 (0.76–4.17)	0.183
Albumin (≥3.5 g/dL vs. <3.5 g/dL)	2.47 (1.44–4.25)	0.001	3.16 (1.18–7.65)	0.016
Crp (≤10 mg/L vs. >10 mg/L)	2.13 (1.35–3.37)	0.001	1.48 (0.80–2.73)	0.202
LIPI group		0.008		0.048
(Good vs. intermediate)	1.56 (1.05–2.31)	0.027	1.59 (0.79–3.18)	0.188
(Good vs. poor)	2.15 (1.30–3.55)	0.003	2.53 (1.32–6.38)	0.034
GPS (0 vs. 1–2)	2.00 (1.26–3.17)	0.003	0.79 (0.41–1.54)	0.499
PNI (high vs. low)	1.70 (1.14–2.54)	0.009	1.32 (0.72–2.40)	0.361
NPS (0 vs. 1–2)	1.67 (1.17–2.39)	0.004	0.74 (0.37–1.50)	0.414

Footnote: HR, hazard ratio; CI, confidence interval; ECOG PS, Eastern Cooperative Oncology Group performance status; PD-L1 TPS, programmed death-ligand 1 tumor proportion score; CRP, C-reactive protein; LIPI, Lung Immune Prognostic Index; GPS, Glasgow Prognostic Score; PNI, prognostic nutritional index; NPS, neutrophil–platelet score. OS, overall survival.

**Table 8:** Comparison of the prognostic performance of inflammation-based scores.

Score	AUC (95% CI)	Interpretation
LIPI	0.605 (0.529–0.681)	Moderate discriminatory ability
GPS	0.576 (0.485–0.667)	Modest discriminatory ability
PNI	0.560 (0.470–0.651)	Modest discriminatory ability
NPS	0.549 (0.471–0.627)	Poor discriminatory ability

Footnote: Abbreviations: AUC, area under the curve; CI, confidence interval; LIPI, Lung Immune Prognostic Index; GPS, Glasgow Prognostic Score; PNI, prognostic nutritional index; NPS, neutrophil–platelet score. AUC values were derived from receiver operating characteristic (ROC) curve analysis to assess the discriminatory ability of each prognostic score.

## Data Availability

The original contributions presented in this study are included in the article. Further inquiries can be directed to the corresponding author.
